# Successful Endovascular Treatment of Iatrogenic Thyrocervical Trunk Pseudoaneurysm with Concomitant Arteriovenous Fistula Using 0.010-Inch Detachable Microcoils

**DOI:** 10.1155/2014/479656

**Published:** 2014-12-24

**Authors:** Kohei Hamamoto, Mitsunori Nakano, Kiyoka Omoto, Masahiko Tsubuku, Emiko Chiba, Tomohisa Okochi, Katsuhiko Matsuura, Osamu Tanaka

**Affiliations:** ^1^Department of Radiology, Saitama Medical Center, Jichi Medical University, 1-847 Amanuma-cho, Omiya-ku, Saitama 330-8503, Japan; ^2^Department of Cardiovascular Surgery, Saitama Medical Center, Jichi Medical University, 1-847 Amanuma-cho, Omiya-ku, Saitama 330-8503, Japan; ^3^Department of Laboratory Medicine, Diagnostic Ultrasound Division, Saitama Medical Center, Jichi Medical University, 1-847 Amanuma-cho, Omiya-ku, Saitama 330-8503, Japan; ^4^Department of Radiology, Maruyama Memorial General Hospital, 2-10-5 Hon-cho, Iwatsuki-ku, Saitama 339-8521, Japan

## Abstract

Pseudoaneurysms (PsA) and arteriovenous fistulae (AVF) of the thyrocervical trunk and its branches are rare complications of traumatic or iatrogenic arterial injuries. Most such injuries are iatrogenic and are associated with central venous catheterization. Historically, thyrocervical trunk PsA and AVF have been managed with open surgical repair; however, multiple treatment modalities are now available, including ultrasound-guided compression repair, ultrasound-guided thrombin injection, and endovascular repair with covered stent placement. We report a case of a 65-year-old woman with an iatrogenic thyrocervical trunk PsA with concomitant AVF that developed after attempted internal jugular vein cannulation for hemodialysis access. The PsA was successfully treated by transcatheter coil embolization using 0.010-inch detachable microcoils. Our case is the first published instance of a thyrocervical trunk PsA with concomitant AVF that was successfully treated by endovascular procedure.

## 1. Introduction

Pseudoaneurysms (PsA) and arteriovenous fistulae (AVF) of the thyrocervical trunk and its branches are rare complications of traumatic or iatrogenic arterial injuries. Most of these injuries are caused by iatrogenic needle puncture of the thyrocervical trunk at the time of attempted internal jugular vein catheterization. The typical clinical symptom of PsA is a pulsatile mass with bruit at the injured site, while the typical clinical symptoms of AVF are bruit and cardiac insufficiency [1–14]. A PsA usually requires intervention because of the risk of complications including pain, a mass effect involving adjacent structures, and rupture; in contrast, an AVF infrequently requires intervention because it often resolves spontaneously [15].

Historically, thyrocervical trunk PsA and AVF have been managed with open surgical repair. In recent years, however, endovascular interventional procedures have often served as the first-line therapy for PsA and AVF in other locations, such as the femoral artery [16]. Although several cases of successful treatment by percutaneous and endovascular procedures have also been reported in the thyrocervical trunk region [9, 11, 12, 14], most of these reports described branched lesions of the thyrocervical trunk or solitary lesions without AVF. Therefore, this approach to cases involving main trunk lesions or concomitant PsA and AVF remains challenging. We herein describe a case of an iatrogenic thyrocervical trunk PsA with concomitant AVF involving the internal jugular vein after central venous catheter insertion for hemodialysis. Successful coil embolization of the PsA and AVF was achieved using 0.010-inch detachable microcoils.

## 2. Case Report

**Figure 1:**
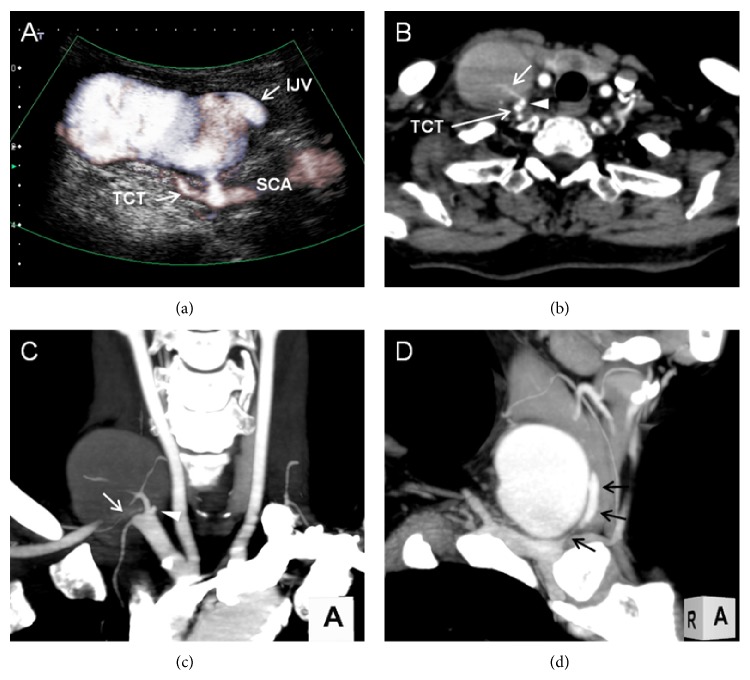
(a) Duplex ultrasound in the longitudinal plane at the level of the right supraclavicular fossa. The PsA communicated with the root of the thyrocervical trunk (TCT) with inner turbulent flow via the short, narrow neck. Continuity between the PsA and the internal jugular vein (IJV) was also noted. SCA: subclavian artery. (b) Contrast-enhanced computed tomography angiography showed a high-density-flow jet of contrast agent shunting from the TCT (arrow) into the aneurysmal sac via the short neck (arrowhead). (c) Maximum-intensity projection (MIP) image in the arterial phase clearly showed the anatomical relationship between the TCT and PsA neck (arrowhead). A flow jet within the PsA was also shown (arrow). (d) MIP image in the venous phase showed continuity between the aneurysmal sac and internal jugular vein (arrows).

**Figure 2:**
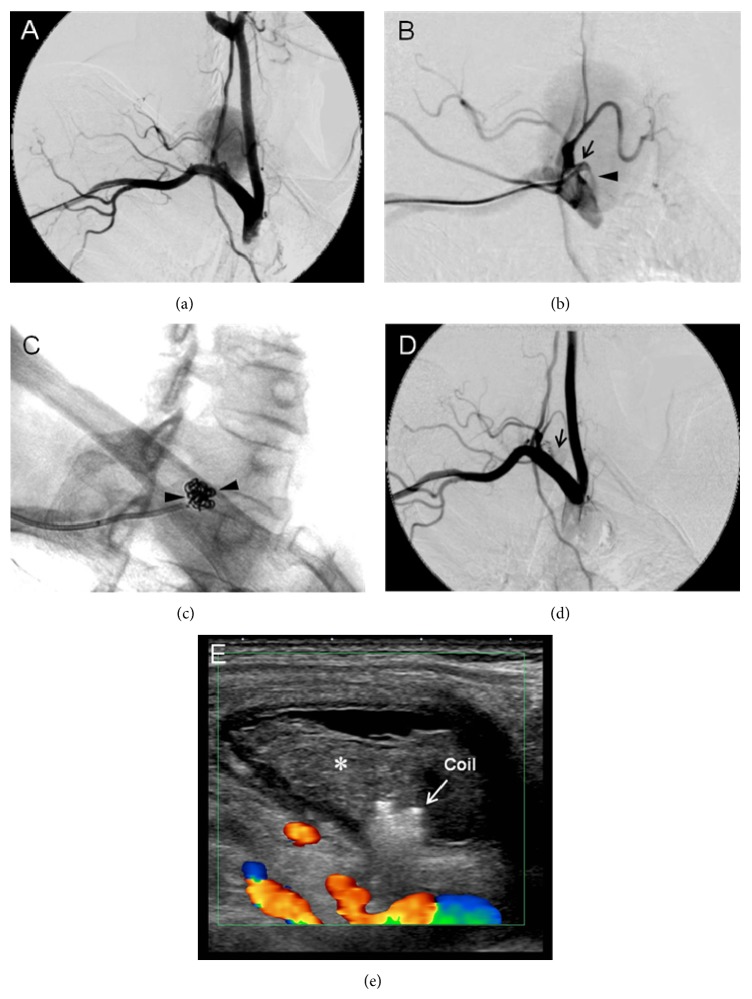
(a) Digital subtraction angiogram (DSA) in the right anterior oblique view showed a large PsA originating from the TCT. (b) Selective DSA of the PsA neck clearly showed the short narrow neck originating from the proximal side of the TCT root. (c) Coil embolization of the PsA. Arrowheads indicate the coils corresponding to the PsA neck. (d) DSA after coil embolization showed the near disappearance of the PsA, but faintly residual contrast dye was noted near the coil (arrowhead). (e) Duplex ultrasound following 15 min ultrasound-guided compression. Complete disappearance of blood flow within the PsA and thrombus formation (asterisk) was observed.

A 65-year-old woman was referred to our interventional radiology department for investigation of a palpable mass and irritating audible bruit on her right neck. Two months prior to this visit, she had developed an acute exacerbation of chronic renal failure secondary to viral gastroenteritis and underwent temporary hemodialysis using a central venous catheter placed in her right jugular vein. Upon arrival at our hospital, a 50 × 30 mm soft mass was palpable over the right side of her neck, and systolic bruit was heard during auscultation. Laboratory data showed elevated levels of serum creatinine and blood urea nitrogen at 155.6 *μ*mol/L and 10.0 mmol/L, respectively. Duplex ultrasonography evaluation of the neck was performed, which showed a 48 × 27 × 46 mm aneurysmal sac that connected the craniomedial side of the thyrocervical trunk via a short narrow neck approximately 3 mm in length and 2.5 mm in diameter (Figure 1(a)). The aneurysmal sac also continued to the internal jugular vein, and the cranial aspect of the internal jugular vein was occluded by a thrombus. Computed tomography angiography of the neck showed a high-density-flow jet of contrast agent shunting from the thyrocervical trunk into the aneurysmal sac via the short neck in the arterial phase (Figures 1(b) and 1(c)). Continuity between the aneurysmal sac and internal jugular vein was also noted (Figure 1(d)). Based on these findings, the diagnosis of iatrogenic PsA of the thyrocervical trunk with an AVF involving the internal jugular vein was made. We discussed surgical versus endovascular intervention with the patient, and the latter was elected. A 4-Fr sheath (Terumo, Tokyo, Japan) was inserted through the right brachial artery, and the right subclavian artery was selectively catheterized by a 4.2-Fr Judkins Right 4.0 angiographic catheter (Goodman, Nagoya, Japan). Selective digital subtraction angiography of the right subclavian artery showed a PsA originating from the thyrocervical trunk root; the PsA had a short narrow neck that connected it to the internal jugular vein (Figures 2(a) and 2(b)). Based on the angiographic findings, we decided to perform coil embolization of the PsA neck because this method was likely to reduce the blood flow of the PsA while preserving the flow of the subclavian artery and other branches of the thyrocervical trunk. Using the abovementioned Judkins Right catheter and a 0.035-inch hydrophilic wire (Radifocus; Terumo, Tokyo, Japan), selective catheterization of the ostium of the PsA neck was achieved. A 2.0-Fr microcatheter (Excelsior1080; Stryker Neurovascular, Fremont, CA, USA) was then introduced coaxially over a 0.016-inch-diameter guide wire (Meister; Asahi Intecc, Nagoya, Japan) and placed into the proximal site of the PsA via its neck. Four 0.010-inch detachable coils (Target Helical Ultra; Stryker Neurovascular) measuring 3.0 mm × 8.0 cm, 2.5 mm × 6.0 cm, 2.0 mm × 6.0 cm, and 2.0 mm × 4.0 cm were sequentially placed across the PsA neck (Figure 2(c)). Subclavian angiography after coil embolization revealed faintly residual flow of contrast medium in the sac, but the flow volume was obviously reduced (Figure 2(d)). The blood flow of the thyrocervical trunk and its branches had been preserved. Additional coil placement was considered to be likely to achieve complete occlusion but possible undesirable embolization of the thyrocervical trunk. Ultrasonographic reevaluation revealed thrombus formation and an obvious decrease in the shunting flow within the PsA. Additionally, the diameter of the PsA was reduced. We determined that the coil embolization was effective, and ultrasound-guided compression repair was performed for 15 min to achieve complete occlusion. Finally, the turbulent flow in the aneurismal sac completely disappeared (Figure 2(e)), and the procedure was thus finished. Ultrasonography performed the next day showed a reduction in the size of the PsA with thrombolization. The patient was discharged 3 days after embolotherapy without worsening of renal function. In the reevaluation of 6 months after embolization, no mass or bruit was detected on her neck. Duplex ultrasonography showed complete thrombolization of the aneurysmal sac with partial cystic change. The size of the aneurysmal sac had been further reduced to 10 × 11 × 8 mm.

## 3. Discussion

In the present report, we describe a case of iatrogenic thyrocervical trunk PsA with concomitant AVF involving the internal jugular vein that was successfully treated by transcatheter coil embolization. Although PsA and AVF are well-documented complications of traumatic and iatrogenic arterial injuries, the occurrence of this condition in the thyrocervical trunk region is rare, as this structure is well protected and is located deep in the neck. Following a literature search performed using PubMed, we noted that only thirteen cases of PsAs [1, 3, 5–11, 14] and five cases of AVFs [2, 4] that were successfully treated with surgical repair or endovascular procedures have been reported (Table 1). To our knowledge, there is no report in the literature of a case with the coexistence of these conditions or a main trunk PsA that was successfully treated with an endovascular procedure.

The PsAs and AVFs of the thyrocervical trunk and its branches are usually iatrogenic in origin and are presumably caused by needle puncture of the thyrocervical trunk at the time of attempted catheterization of the internal jugular vein. A reported risk factor for iatrogenic PsA of the thyrocervical trunk is the use of a lower or lateral approach to the internal jugular vein [1]. Overanticoagulation, age, atherosclerosis, hypertension, and anatomical variation of the internal jugular vein are additional risk factors of central venous catheterization-related PsA [17]. Several treatment options are available for PsA and AVF, including surgical resection, ultrasound-guided compression (USGC), ultrasound-guided thrombin injection (UGTI), and endovascular procedures such as stent placement and embolization by using a metallic coil or vascular plug. In recent years, endovascular interventional procedures have often served as the first-line therapy as these methods are less invasive.

The treatment of PsAs and AVFs of the thyrocervical trunk is dependent on their location, anatomical form of the PsA neck or AVF tract, and the presence or absence of these coexisting conditions. In cases with solitary and branch lesions, relatively simple percutaneous or endovascular procedures such as USGC, UGTI, and coil embolization of culprit arteries can be employed [9–12, 14]. However, the occurrence of PsAs located at the root of the thyrocervical trunk and AVF, as in the present case, is believed to be more complex, and there is no established treatment. Historically, PsAs developing in this region have been managed with open surgical repair [1, 3, 5–8, 10]. Although USGC and USTI are less invasive techniques, they may be difficult to apply in cases with main trunk lesions. USGC, by itself, cannot achieve hemostasis, because the root of the thyrocervical trunk is located deep within the soft tissue of the neck. Moreover, UGTI is associated with an increased risk for systemic thrombosis or embolization in cases where the PsA originates from the adjacent area of the thyrocervical trunk root or is accompanied by AVF [18].

The closure of peripherally located PsAs and AVFs using detachable coils is a well-established and effective procedure; this was believed to be the most suitable treatment in the present case, as specific embolic materials can be specifically chosen to control the embolization. In the present case, a short-segment embolization of the PsA neck was required to preserve the flow in the other branches of the thyrocervical trunk while avoiding coil migration to the venous circulation; hence, we used 0.010-inch detachable microcoils. These coils are thin and highly flexible, and coil movement can be easily controlled by slowly pushing the delivery wire back and forth. This enabled the placement of tightly overlapping coils during coil embolization, thus facilitating the treatment of the short segments of small target vessels even in the critically limited area of the embolization. Although USGC was also needed, we achieved complete blood flow occlusion of the PsA and AVF without any unintended embolization of nontargeted vessels or coil migration into the venous circulation.

Other materials can also be used for PsA and AVF occlusion, such as the Amplatzer vascular plug or N-butyl cyanoacrylate glue. However, both approaches were believed to be unsuitable in the present case because of anatomical complexity of the PsA neck and coexistence of AVF. Covered or uncovered stent placement in the thyrocervical trunk is another alternative endovascular procedure; however, it is not an ideal procedure because of the risk of restenosis. Additionally, patients should undergo long-term treatment with antiplatelet drugs to prevent restenosis.

## 4. Conclusion

Thyrocervical trunk PsA and AVF following central venous catheterization are a rare complication and can manifest days to months after the initial injury. Transcatheter coil embolization is a safe and effective treatment for PsA, even in cases accompanied by AVF or main trunk lesions. This is the first report of transcatheter coil embolization of a thyrocervical trunk PsA with concomitant AVF involving the internal jugular vein.

## Figures and Tables

**Table 1:** Chart review of pseudoaneurysm and arteriovenous fistula of thyrocervical trunk.

Reference	Side	Location	Age	Etiology	Symptoms	Time to onset of symptoms after injury	Treatment	Outcome
Thyrocervical trunk pseudoaneurysms								
Shield III et al. [1]	R	Main trunk	54	Iatrogenic	Pulsatile mass	3 months	Surgical resection	Successful
	R	Main trunk	16	Iatrogenic	Nonpulsatile mass	6 weeks	Surgical resection	Successful
den Hollander and Slapak [3]	R	Main trunk	16	Iatrogenic	Pulsatile mass with bruit	5 weeks	Surgical resection	Successful
Abrokwah et al. [5]	R	Main trunk	78	Iatrogenic	Horner's syndrome	1 day	Surgical resection	Successful
Elariny et al. [6]	R	Main trunk	78	Iatrogenic	Pain, bruit	2 days	Surgical resection	Successful
Houshian and Poulsen [7]	R	Main trunk	26	Traumatic	Pulsatile mass with bruit	4 months	Surgical resection	Successful
Peces et al. [8]	R	Main trunk	57	Iatrogenic	Pulsatile mass with bruit	3 months	Surgical resection	Successful
Majeski [10]	R	Main trunk	36	Traumatic	Pain, pulsatile mass	2 months	Surgical resection	Successful
Cuhaci et al. [9]	R	Branch	46	Iatrogenic	Pain, pulsatile mass	2 weeks	Coil embolization	Successful
Ramsay and McAuliffe [11]	L	Branch	36	Traumatic	Pain, pulsatile mass	4 hours	Coil embolization	Successful
Dwivedi et al. [12]	R	Branch	67	Iatrogenic	Pain, pulsatile mass	2 days	Coil embolization	Successful
Mazzei et al. [13]	R	Branch	71	Iatrogenic	Pulsatile mass	3 months	Surgical resection	Successful
Mehta et al. [14]	R	Branch	56	Traumatic	Pain, pulsatile mass	4 months	UGTI	Successful

Thyrocervical trunk arteriovenous fistula								
Glaser et al. [2]	R	Main trunk	53	Iatrogenic	Chronic heart failure	2 months	Surgical resection	Successful
Herbreteau et al. [4]	R	N.D.	N.D.	Iatrogenic	Bruit, cardiac insufficiency	N.D.	Occluded by DBC	Successful
	R	N.D.	N.D.	Iatrogenic	Bruit	N.D.	Occluded by DBC	Successful
	R	N.D.	N.D.	Iatrogenic	None	N.D.	Occluded by DBC	Successful
	R	N.D.	N.D.	Iatrogenic	Bruit, chronic heart failure	N.D.	Occluded by DBC	Successful

N.D.: not described precisely; R: right; DBC: detachable balloon catheter; UGTI: ultrasound-guided thrombin injection.
